# Primary care for persons living with dementia by Nurse Practitioners: practice management, organizational context, and job outcomes

**DOI:** 10.1002/alz70858_103523

**Published:** 2025-12-24

**Authors:** Lusine Poghosyan, Kyle G Featherston, Maura Dougherty, Grant Martsolf, Josh Porat‐Dahlerbruch, Soo Borson, Tatiana Sadak, Siqing Wang, Monica O'Reilly‐Jacob

**Affiliations:** ^1^ Columbia University School of Nursing, New York, NY, USA; ^2^ University of Pittsburgh School of Nursing, Pittsburgh, PA, USA; ^3^ University of Washington, Seattle, WA, USA; ^4^ Yale University School of Nursing, Orange, CT, USA

## Abstract

Delivering care to the more than 55 million people living with dementia (PLWD) worldwide is critically important, and healthcare systems search for innovative solutions to increase the primary care system's capacity to care for these patients. Advanced practice nurses such as nurse practitioners (NPs) are vital to increasing the primary care capacity to meet the demand for dementia care. Yet, primary care NPs often face structural, organizational, and workforce challenges. This study explored the practice management capabilities, organizational context, and job outcomes (i.e., burnout, job dissatisfaction, and intent to leave the practice) among NPs in the U.S. providing care for patients with dementia in primary care practices.

We conducted a national survey of NPs using modified Dillman methods. NPs who cared for 30 or more PLWD and other NPs at their practices received mail and online surveys between 2021‐2023. In total, 968 NPs responded across 847 practices. We estimated a response rate between 16.4‐36.4%, depending on assumptions about whether non‐respondents were eligible for the study. NPs reported significant deficits in practice management for PLWD care compared to other chronic conditions such as cardiovascular diseases, diabetes, and depression Specifically, fewer than 60% of NPs reported tools, templates, and reminders for PLWD care, which were more widely available for the other chronic conditions. Additionally, NPs reported poorer quality of care for dementia compared to overall care and challenges with administration within their organization. The findings show that almost 36% of NPs were burned out and over one‐fifth reported they intended to leave their jobs within the next year. Having specific tools, templates, and reminders for PLWD was modestly correlated with lower dementia care ratings (*r* = ‐.26), as well as NP burnout (*r* = ‐.17).

Given the projected growth in the number of PLWD, policy and practice efforts should be directed towards strengthening primary care practices to provide quality care for dementia patients. A widespread burnout among these NPs raises concerns about patient care and workforce retention. Bolstering the capacity of the NP workforce and supporting their practice within organizations can ensure a dementia‐capable workforce and improve patient outcomes.

